# Knowledge, Perception and Preparedness Related to COVID-19 among Adult Rural Population in Konso, Southern Ethiopia

**DOI:** 10.4314/ejhs.v32i1.2

**Published:** 2022-01

**Authors:** Girma Gilano, Serawit Lakew, Tadiwos Hailu, Be'ement Tekabe, Sewunet Sako, Tesfaye Feleke

**Affiliations:** 1 Department of health informatics school of public health, Arba Minch University College of Health Science Nech Sar campus, South West Ethiopia

**Keywords:** Knowledge, Perception, preparedness, COVID-19, Konso

## Abstract

**Background:**

According to the world health organization, the COVID-19 outbreak has currently affected millions of people worldwide. Since the start of the pandemic in China, everything countries have thrown at the virus turned unsuccessful. As there is no established vaccine to halt the virus transmission, it might be very difficult for developing countries like Ethiopia even after vaccine development. Thus, focusing on improving knowledge, perception, and preparedness related to the virus might be very vital.

**Methods:**

A community-based cross-sectional survey was conducted using the questionnaire applied in most countries which is suitable to apply in the Konso zone in South Nations Nationalities Region (SNNPR) from April.2020 to July 2020. Data were collected, cleaned, coded, checked, and entered into Epi Info and then exported and analyzed in STATA 15. We fitted a binary logistic regression model. Categorical variables were presented using figure tables, and proportion and for continuous variables, mean and standard deviation were used. The results were also presented using Adjusted Odds Ratio (AOR) with 95% CI.

**Results:**

From 636 respondents expected, 615(97%) were participated and possessed the knowledge of 39%, and 64% perception and preparedness each. Measurements related to the policy and interventions like restrictions to movement, self-isolation, wearing a face mask, & the need for more tests was not supported by most participants.

**Conclusion:**

The poor knowledge, perception, and preparedness were correlated with the unavailability of water and electricity, less education, and informal source of information which could be improved through area appropriate health education interventions.

## Introduction

Since 31 December 2019, rapidly spreading grave pneumonia cases of anonymous agents were conveyed to the attention of the World Health Organization (WHO). It was then confirmed as the novel coronavirus (SARS-CoV-2) and declared to be a global public health emergency by the WHO on January 30, 2020(1). By what was termed as super spreading in March, many thousand cases were testified in over hundred countries. Since then quarantine and restriction were being issued(2). However, there was no method worked very well against the virus, thus, personal protection equipment, social distance, and movement restriction got major focus (3). Some sources predicted the outbreak might last 18 to 24 months with the possibility of the 60% to 70% infection in the general population that enquires real preparation (4). Missing the opportunity to increase public awareness and perception that might enable protecting self and others could not be afforded (5).

Studies linked good knowledge and preventive practice or preparedness related COVID-19 with the prevention or minimization of future waves of the pandemic (6). There are also other studies which indicated that the gap that might exist in the public regarding knowledge, attitude, and practice might be costly in terms of live loss (7). There are much worrying results which show negative perception of people regarding contracting the virus - some people think they cannot contract the virus because it is a political propaganda while others think their religion can protects them without themselves complying with the protocols (8). There are evidences indicating that the impact of the pandemic will remain severe on economically under developed countries and areas where cultural factors and under education might affect preventive activities (9). Much evidence agrees that COVID-19 is just more than a health crisis; it is an economic and social crisis. The fact is very different in already under developed countries. It might cause painful scene, if preventive activities are not intensively (awareness creation, perception, and preventive practices) included in strategies (10).

Recommendation from some studies indicated that adequate time for awareness creation and working for good perception could be the only weapon against the coronavirus disease 2019(COVID-19) (11). This was reflected in the United States (US) survey, where people know the threat but still do not think they will be infected with 2019-nCoV (12). This appeals countries for exhaustive awareness creation and strong solidarity in the joint efforts to fight COVID-19 dissemination(13). The solution was indicated in other studies to be training on self and community protection measurements which encompasses individual educational attainment and believed to be effective in Ethiopia; however, there was enough reason to undertake further studies(14).

A study in Nigeria, Kenya, and South Africa showed high knowledge better than that was in Ethiopia which was only 25% (15–17). Studies claimed that such problems might be tackled through creating a chain of communication among providers, school students, and non-educated individuals so that governments could contain the pandemic (18). Further, increasing the trust among the public and institutions could do a better job to control the virus (19). The other mandatory assertion from studies was protecting populations at risk of developing all types of complications, which is also impossible without good awareness, perception, and preparedness (20). Indeed, a health education paradigm targeting awareness, perception and preparedness toward COVID-19 was provided as a tool in literature(21). During the current study there was less cases in area compared to other parts of the world; however, uncontrollable community transmission was always feared considering the type of settlement and way of life in the area (22). This means there was an opportunity not to miss in Ethiopia and possibly in sub-Saharan countries while cases remain small(23). Evidence indicates, China, Europe, the US, and other countries consequently paid a huge cost which would have been otherwise reduced (5). From the study conducted on visitors of Jimma University hospital, there is clear evidence that indicated the current knowledge and preventive practices were not adequate enough to combat the pandemic (24). However, this doesn't mean that everything is similar throughout the country. To make implementation of preventive practices intensively where it is necessary and put focus on high-risk areas, studies like this are very crucial. Therefore, this study was aimed to identify knowledge, perception, and preparedness related to COVID-19 among the rural community in Konso zone karat Zuria woreda to prepare the community and also bring the level of understanding and preparedness in to the government attention.

## Methods

**Participants and design**: **A** community-based cross-sectional survey was conducted to assess the level of knowledge, perception, and preparedness related to COVID-19 among the adult population in the Konso zone Karat zuria woreda in South Nations Nationalities region from April.2020 to July 2020. Konso is a unique place in rural southern Ethiopia. People of Konso were known by terracing, unique cultural identities, and specially gifted landscapes registered as a UNESCO World Heritage Site since 2011.

The study included the adult population living in randomly selected kebeles in the woreda which who are not aged <18, not unable to communicate with data collectors due to the serious illness, resided 6 month and above in the area, and those who signed the consent of participation and willing to follow WHO COVID-19 protection protocols during the data collection. Data protection, privacy, benefit, and refusal right all explained for participants. A single proportion of population formula was applied considering the proportion of knowledge, perception, and preparedness of coronavirus 2019 among the community 50% with addition of 10% non-response rate and multiplying with 1.5 design effect to get 636 people. Karat Zuria woreda has 14 kebeles which are at different distance from the Karat town. Each kebele is a separate and distinct cluster. Since we selected four kebeles randomly according to the WHO protocol and also followed another selection procedure within the households when the number of adults in a household is greater than one, it was necessary to apply design effect.

**Variables of the study**: The outcome variables of the study were knowledge, perception, and preparedness, while exploratory variable were sex, age in year, marital status, educational status, source of info, medical checkup, chronic illness, monthly income, religion, occupational status, alcohol drinking, and presence of electricity

**Measurements**: Socio-demographic measurements; Age: treated as continues; Marital status:1_single, 2_married, 3_divorced & 4_widowd, 5_separated; educational status: 1_illiterate, 2_read and write, 3_primary cycle, 4_high school, 5_certificate & 6_diploma and above; religion: 1_protestant, 2_Orthodox & 3_muslims; occupational status: 1_spouse, 2_farmer, 3_marechant, 4_student & 5_gov't employee; number of people in a house and monthly income were taken as continues and all the others remaining variables in this category were measured as yes/no.

**Knowledge**: Knowledge is one's awareness of health risks of COVID-19 including signs and symptoms of the disease. Knowledge as a dependent variable was measured by 24 items focusing on self-assessment, signs and symptoms, treatment/vaccination, incubation, and preventions of the novel coronavirus infection. Each category was scored as ‘yes (1)’, ‘no (2)’, & ‘I don't know (3)’. The knowledge scoring range of the data collection tool was 24 (best to worst). Scores then coded ‘1’ and all else as ‘0’ for the presence and absences of necessary factors respectively. All definitions were taken according to the WHO European region CDC guideline (25).

**Perception**: refers to the way sensory information is organized, interpreted, and consciously experienced. It is the way a person thinks about how to prevent and how he or she interprets information related to these activities. Perception in this study was measured by 25 items: trust in institution, policies & interventions, risk perception/probability & severity, trust in sources of information, and resilience. Trust in institutions, included 6 items and was measured with a five-point liker scale as 1-Very low confidence, 2- low confidence, 3- undecided, 4- confident, & 5- Very high confidence. Policy & intervention and Perceived severity consist of another 10&3 items that were measured using the five-point Likert scale as 1_strongly disagree, 2-disagree, 3-undecided, 4-agree, & 5- strongly agree each. Trust in sources of information captured with 3 items measured on the same scale as 1-not certainly true, 2-not true, 3-undecided, 4-certainly, and 5-certainly true. While the remaining 3 items under resilience were measured as 1- strongly disagree, 2-disagree, 3-undecided, 4-agree & 5-strongly agree. Finally, the scales were considered as continuous. The average 3.5 was considered as a cut point so that ≥3.5 was coded as ‘1’ and all else were coded as ‘0’ which allowed us to understand perception, and trends related to this & are useful for planning communications and detecting possible shifts in perception (e.g. following certain events or new restrictions) which can inform/promote/avoid future events. It is useful to stratify risk perceptions, population groups, knowledge, and others.

**Preparedness**: Taking control of any incident, emergency, or crisis and having a plan for the next event during emergency situation. Preparedness and perceived self-efficacy: measured with 14 items on the five-pointed Likert scale as 1-strongly disagree, 2-disagree, 3-undecided, 4-agree & 5-strongly agree. This allowed us to understand readiness and community responses (25).

**Data management and analysis**: An Amharic translated questionnaire was adapted from the survey guidance of rapid, simple, and flexible behavioral insight instruments on COVID-19 which were originally developed by the WHO European region CDC (23) in parallel with considerable tools in the literature (6, 17) Trained data collectors and supervisors were employed to collect the data after pre-testing the tool. The Cronbach's alphas for Knowledge, perception, and preparedness were 0.847, 0.847, and 0.870 respectively. Data was processed, entered into EPI Info, transferred, and analyzed in STATA 15. Means, standard deviation (mean ±SD) after checking normality by presenting continuous data using histogram, and percentages were also used to describe the data. We included variables in the model at p-value <0.25 during bivariate analysis and *p*-values <0.05 was used to declare association multivariate analysis with 95% CI. Compared to the null model, the final model showed a significant improvement and was used to fit the data. Multicolinearity was checked and found to be 3.9 which is in the safe range. The binary logistic regression was used to see the association between the dependent and independent variables. Ethical clearance according to the nature of the COVID-19 prevention protocol was obtained from the Institutional Review Board of Arba Minch University.

## Results

**Descriptive statistics**: From the 636 respondents approached, and the response rate was 97%. The socio-demographic descriptions of the participants are displayed in Table 1.

**Table 1:** The socio-demographic characteristics of respondents at Karat Zuria woreda in 2020 (N=615)

Variables	N (%)
Sex	
Male	306(49.80)
Female	309(50.20)
Age in year	
≤20	130(21.10)
21–30	195(31.70)
31–40	99(16.10)
41–50	102(16.60)
≥51	89(14.50)
Marital status	
Single	187(30.4)
Married	418(68.0)
Divorced	10(1.60)
Educational status	
Illiterate	332(54.0)
Read and write	26(4.20)
Primary school	142(23.10)
High school	63(10.20)
Certificate and above	52(8.50)
Source of info	
Radio	131(21.30)
TV	24(3.90)
Peer/neighbor	434(70.60)
Internet	26(4.20)
Medical checkup	
Yes	43(7.0)
No	572(93.0)
Chronic illness	
Yes	67(10.90)
No	548(89.10)
Monthly income	
≤300	223(36.30)
301–600	232(37.70)
601–900	74(12.0)
≥901	86(14.0)
Religion	
Protestant	566(92.0)
Orthodox	25(4.10)
Others	24(3.90)
Occupational status	
Spouse	28(4.60)
Farmer	400(65.0)
merchant	27(4.40)
Student	134(21.80)
Gov't employee	26(4.20)
Presence of electricity	
Yes	170(27.60)
No	445(72.40)
Water availability	
Yes	201(32.70)
No	414(67.30)
Alcohol drinking	
Yes	221(35.90)
No	394(64.10)

Over half (52%) of the participants were aged 30years or below. More than two-thirds were married (68%) and half of them were illiterates (54%). Occupationally, around two-third were farmers and less than 5% were government employees. The income for most participants was ≤600 (74%). The mean household size was 6.7±3.1. The source of information for participants was Peers/neighborhoods (70%) and only 27.6% reported presence of electricity in their house.

**Figure 1:**
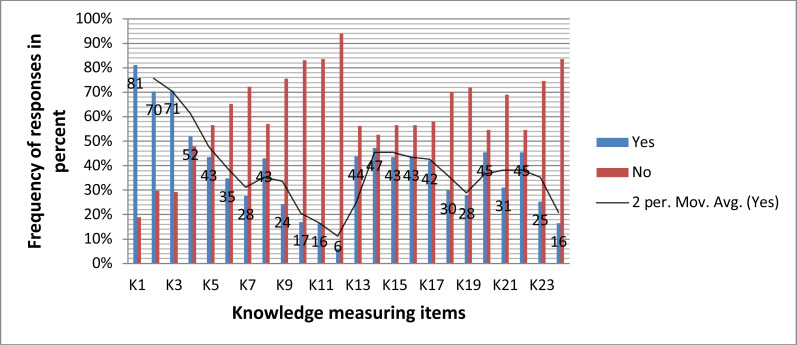
Distribution of the knowledge related about COVID-19 among adult people in Karat Zuria woreda, South Ethiopia in 2020 (N=615)

**Description of knowledge, perception and preparedness parameters**: The average knowledge status of the participants was only 39%. The respondents had good knowledge in COVID-19 prevention methods (81%) and cough (71%) and fever (70%) as the signs and symptoms of COVID-19 a (Figure 1).

In the Figure 1, K1=know COVID-19 prevention, K2=fever, K3=cough, K4=shortness of breath, K5=sore throat, K6=runny/stuffy nose, K7=muscle ache, K8=headache, K9= fatigue, K10=Diarrhoea, K11=loss of taste/smell, K12=presence of the drug, K13=presence of vaccine, K14=incubation period, K15, authority recommendations, K16=hand washing, K17=touching eye/nose/mouth, K18=staying home, K19=use herbs, K20=cover mouth/nose, K21=wear mask, K22=physical distancing, K23=disinfect surfaces, K24=eat garlic/ginger/lemon.

**Figure 2:**
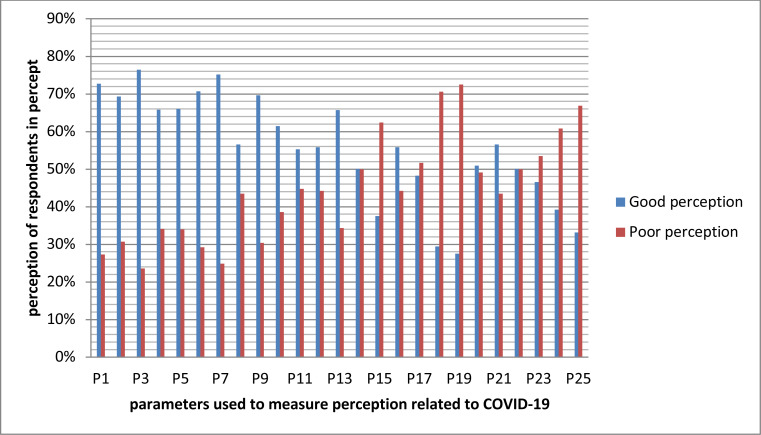
Distribution of the perception related about COVID-19 among adult people in Karat Zuria woreda, South Ethiopia in 2020 (N=615).

The overall average perception of respondents was 64% which corresponds to 3.20±0.81 on a continuous scale. Confidence in professionals (72%), confidence in media reports (71%), confidence in the ministry of health (74%), confidence in local health authority (70%), confidence in gov't decisions (71%), and willingness to take vaccine or treatment when available (73%) were some of the parameters scored good perception (Figure 2).

In the Figure 2, P1=confident of health professionals, P2=confident of media, P3=confident in MOH, P4=confident in health actions, P5=confident in transportation P6=confidence in gov't, P7=take vaccine if any, P8=avoiding certain people, P9=forcing self-isolation, P10=making mask required, P12=More tests, P13= professionals only leave the house, P15= probability of infection, P17=susceptibility, P18=easy recovery, P20=not telling us about others, P21= Politicians tell us false, P22=secret events lack connection, P23=hard time with stress, P24 =easy recovery

The average preparedness of participants was 3.28±0.91 and the overall preparedness of the participants was 64% in proportion. Among items used to measure preparedness self-protecting (73%) and practicing preventive action everyday (70%) were scored higher.

**Analyses of associated factors with knowledge, perception, and preparedness**: The odds of knowledge related to COVID-19 becomes higher as age increased by a year with AOR of 0.98(0.97, 0.999), but this worth nothing as the difference was only 2%. Participants who were Illiterate and those who have primary cycle education had 88% and 84% reduced knowledge about COVID-19 with AOR of 0.12(0.038, 0.38) and 0.16(0.053, 0.53) respectively compared to those educated college and above. Single and married participants had 85% and 81% reduced knowledge of coronavirus relative to the divorced individuals with AOR of 0.15(0.028, 0.82) and 0.19(0.037, 0.98) respectively. Compared to information obtained from TV, peer information had the reduced odd of knowledge about COVID-19 by 43% with AOR of 0.57(0.35, 0.92). Large families were more likely to have good knowledge with AOR of 1.10(1.02, 1.13). similarly, those participants who had medical checkups and those who had water for daily usage had increased odds of good knowledge with AOR of 4.7(2.1, 10.9) and 1.5(1.5, 2.17) respectively (Table 2).

**Table 2:** Association of respondents' Knowledge by their socio-demographic variables (N=615)

Variable	*p-value*	*OR*	*Lower 95%*	*Upper 95%*
Age	0.035	0.98	0.97	0.999
Educational status				
Illiterate	0.000	0.12	0.038	0.38
Read and write	0.08	0.28	0.07	1.20
Primary cycle	0.002	0.16	0.05	0.53
High school	0.42	0.59	0.16	2.11
Certificate	0.55	0.56	0.08	3.80
diploma and above	1.00			
Marital status				
Single	0.048	0.15	0.028	0.82
Married	0.029	0.19	0.037	0.98
Divorced	1.00			
Source of information				
Radio	1.00			
TV	0.18	0.50	0.18	1.38
Peers	0.022	0.57	0.35	0.92
Internet	0.42	1.90	0.389	9.52
Number of households	0.041	1.10	1.02	1.13
Medical checkup	0.000	4.70	2.10	10.90
Water availability	0.044	1.50	1.01	2.17

Information from peers were also associated with lower perception with AOR of 0.53(0.29, 0.97) compared to radio information. Participants who had medical checkups, water for daily activities, and those with large families had the higher odds of higher perceptions related to COVID-19 with AOR of 4.50(1.12,21.50), 2.00(1.04, 3.80) and 1.10(1.01, 1.20) respectively; however, drinking alcohol reduced perception of respondents by 55% with the AOR of 0.45(0.26, 0.79) (Table 3).

**Table 3:** Association of respondents' perception with their socio-demographic variables (N=615)

S.N	Variable	*p-value*	*OR*	*Lower 95%*	*Upper 95%*
1	**Source of information**				
	Radio	1.00			
	TV	0.99	0.00	0.00	
	Peers	0.04	0.53	0.29	0.97
	Internet	0.77	1.20	0.41	3.34
2	Chronic illness	0.032	4.50	1.12	21.50
3	Number of households	0.032	1.10	1.01	1.20
4	Drinking alcohol	0.006	0.45	0.26	0.79
	Water availability	0.037	2.00	1.04	3.80

Participants who obtained information from TV and peers had 94% and 66% reduced preparedness toward COVID-19 with AOR of 0.06(0.01, 0.28) and 0.34(0.19, 0.54) compared to internet information respectively. Participants with Illiterate and primary educational status had 75% and 70% reduced preparedness of coronavirus 2019 with AOR of 0.25(0.10, 0.60) and 0.30(0.10, 1.10) respectively compared to those educated diplomas and above. Participants who had electricity and those who had large family were positively associated with preparedness with AOR of 3.02(1.90, 4.70) and 1.10(1.01, 4.66) respectively. in other words, participants who were living with children and those who drink alcohols had 59% and 65% reduced preparedness toward COVID-19 with AOR of 0.41(0.27, 60) and 0.35(0.23, 0.50) respectively (Table 4).

**Table 4:** Association of respondents' preparedness with their socio-demographic variables (N=615)

Variable	*p-value*	*OR*	*Lower 95%*	*Upper 95%*
**Source of information**				
Radio	1.00			
TV	0.000	0.060	0.013	0.28
Peers	0.000	0.34	0.19	0.54
Internet	1.00	0.39	0.13	1.20
**Educational status**				
Illiterate	0.003	0.25	0.10	0.60
Read and write	0.059	0.30	0.10	1.10
Primary school	0.016	0.31	0.12	0.80
High school	0.25	0.54	0.20	1.50
Certificate	0.15	0.29	0.05	1.60
Diploma and above	1.00			
Presence of electricity	0.000	3.02	1.90	4.70
Living with children	0.000	0.41	0.27	0.60
Drinking alcohol	0.000	0.35	0.23	0.50
Number of households	0.035	1.10	1.01	4.66

## Discussion

The knowledge of the participant regarding COVID-19 was not adequate. The poor relationship between self-assessed knowledge and perceived knowledge might be an indication of the information people using. Among many symptoms most respondents only knew fever, cough, and shortness of breath (Figure 1). The finding is consistent with a study in Nairobi, Kenya (35). However, when this compared to other studies in Nairobi, Malaysia, and China the knowledge of symptoms of the outbreak was very poor which could make the prevention and case tracing more difficult (26, 27). This might indicate poor access to the sources of information and shortage of information provided by skilled professionals. Over 70% participants were used secondary information (peer/neighbor) that was inversely correlated with knowledge of the virus. Indeed, there were studies in Kenya and US (26, 28) consistent with this finding. This might indicate that the understanding of the virus was still poor in some parts of the world. Additionally, medical checkups, presence of electricity and water were highly associated with knowledge of participants (fig.1) while a number of studies were confirmed consistent findings (29, 30). This might indicate the unequal access to health services that caused the differences.

Considering the perception, participants showed confidence on government, health professionals' abilities and some institutions (Table 2, Figure 2). However, misperception was recognized in transportation, self-protection, and restrictions consistently with other studies (31). Large number of the respondents in rural area where road is the problem use motor bikes. Banning motor bike without alternative transportation way might make the community to think against the transportation restrictions. In one of the studies such issues were described as the distrust causing between the government and people (32). Absence of water and electricity, people obtaining information from peers or neighborhood, and illiterate educational status were inversely associated with knowledge, and preparedness to prevent coronavirus. In other studies these factors reduced the peoples' self-protection and agreement with those policies (33). However, some studies described the poor association was due to the inconsistent implementation of policies and interventions (34). They also described as the source of the poor perceptions (6,12,16,31). According to the model of crisis communication, identifying the source of information that people believe in could easy complex problems (35). Drinking alcohol was known to affect body immunity and expose the body to the virus. However, people in the study area think that drinking sour drinks including strong alcohol, eating burning fruit like pepper, ginger, and garlic as a prevention method which might be challenging (Figure 2). Such issues were also observed in other studies where misperception jeopardizes the whole prevention efforts (36,).

Preparedness in other studies had larger scores than in our study (37). The inconsistent might be due to the lack of formal information that prepare people for self-protection(32). As discussed above, illiterate educational status, absence of electricity and water for daily activities, and peer or neighborhood source of information were also negatively association with preparedness. While poor educational status remained the obstacle to access information, it was also obvious that the absence of electricity limited the access to formal information from government and private Medias.

In other words, the mean age of the respondents was 34±15; however, over half of the respondents were <30. This is consistent with the last census conducted in Ethiopia (38). The young age is highly associated with the transmission trend and need clear guidance (6). The larger family size observed in the area compared to national and regional magnitude (39) might make restrictions very difficult (Table 1). The COVID-19 prevention might be very difficult due to the absence of water and electricity as respondents reported. Additionally, the lack of access to formal information might make the people short of basic prevention methods. This means identifying suspicious cases, controlling transmission and seeking care would be delayed because of unawareness of the situation (40).

Overall, the study participants have poor knowledge where the magnitude of perception and preparedness were similarly. Increasing the knowledge related to the pandemic through health education based on the educational status of the community might increase awareness and improve perception which would also improve preparedness. Other than knowledge, perception and preparedness factors like electricity, water, peer or neighborhood-based information source, and illiterate educational status were highly associated with poor knowledge, perception, and preparedness. Providing solar system or generators for the community, provision of water with vehicles during the pandemic period, and increasing coverage of local language radio might be considered for solution. The negative attitude toward transportation restrictions, low educational achievements, and the difference between the perceived and the self-assessed knowledge, might make the policy implementations difficult. Thus, intervention aimed at changing the current status and facilitating the further policy implementation inquired.

The study was conducted during the time of the pandemic that limited some of the data capturing methods like observation; however, there was a highly recommended instrument to collect data in such environments and we applied it safely. There were other limitations like cross sectional nature of data that cannot make causal relationship and restriction of making group discussion. The tool itself was developed by WHO European region CDC that might have some difficulty of fitness despite effortful customization; however, the performance of the tool was checked and found very well by Cronbach's Alpha.

Ethical clearance was obtained from the Institutional Review Board of Arba Minch University and go-ahead permission letters were collected from Zonal and woreda health offices. All information related to voluntary participation, refusal and withdrawal, privacy, benefits, and compensation of participation, risks of participation, procedures, and purpose of the study were explained and clarified. Only residents who agreed and signed the consent participated. Non-participants identifying data were collected. Only data collectors and principal investigators were assessed the collected. Infection prevention protocols forwarded by the federal minister of health and that of WHO were strictly maintain under strict supervision in training and data collection.
